# Stop and Go – Waves of Tarsier Dispersal Mirror the Genesis of Sulawesi Island

**DOI:** 10.1371/journal.pone.0141212

**Published:** 2015-11-11

**Authors:** Christine Driller, Stefan Merker, Dyah Perwitasari-Farajallah, Walberto Sinaga, Novita Anggraeni, Hans Zischler

**Affiliations:** 1 Institute of Anthropology, Johannes-Gutenberg University Mainz, Mainz, Germany; 2 Department of Zoology, State Museum of Natural History Stuttgart, Stuttgart, Germany; 3 Primate Research Center, Bogor Agricultural University, Bogor, Indonesia; 4 Department of Biology, Bogor Agricultural University, Bogor, Indonesia; 5 School of Graduate Studies, Bogor Agricultural University, Bogor, Indonesia; BiK-F Biodiversity and Climate Research Center, GERMANY

## Abstract

The Indonesian island of Sulawesi harbors a highly endemic and diverse fauna sparking fascination since long before Wallace’s contemplation of biogeographical patterns in the region. Allopatric diversification driven by geological or climatic processes has been identified as the main mechanism shaping present faunal distribution on the island. There is both consensus and conflict among range patterns of terrestrial species pointing to the different effects of vicariant events on once co-distributed taxa. Tarsiers, small nocturnal primates with possible evidence of an Eocene fossil record on the Asian mainland, are at present exclusively found in insular Southeast Asia. Sulawesi is hotspot of tarsier diversity, whereby island colonization and subsequent radiation of this old endemic primate lineage remained largely enigmatic. To resolve the phylogeographic history of Sulawesi tarsiers we analyzed an island-wide sample for a set of five approved autosomal phylogenetic markers (ABCA1, ADORA3, AXIN1, RAG1, and TTR) and the paternally inherited SRY gene. We constructed ML and Bayesian phylogenetic trees and estimated divergence times between tarsier populations. We found that their arrival at the Proto-Sulawesi archipelago coincided with initial Miocene tectonic uplift and hypothesize that tarsiers dispersed over the region in distinct waves. Intra-island diversification was spurred by land emergence and a rapid succession of glacial cycles during the Plio-Pleistocene. Some tarsier range boundaries concur with spatial limits in other taxa backing the notion of centers of faunal endemism on Sulawesi. This congruence, however, has partially been superimposed by taxon-specific dispersal patterns.

## Introduction

**Fig 1:**
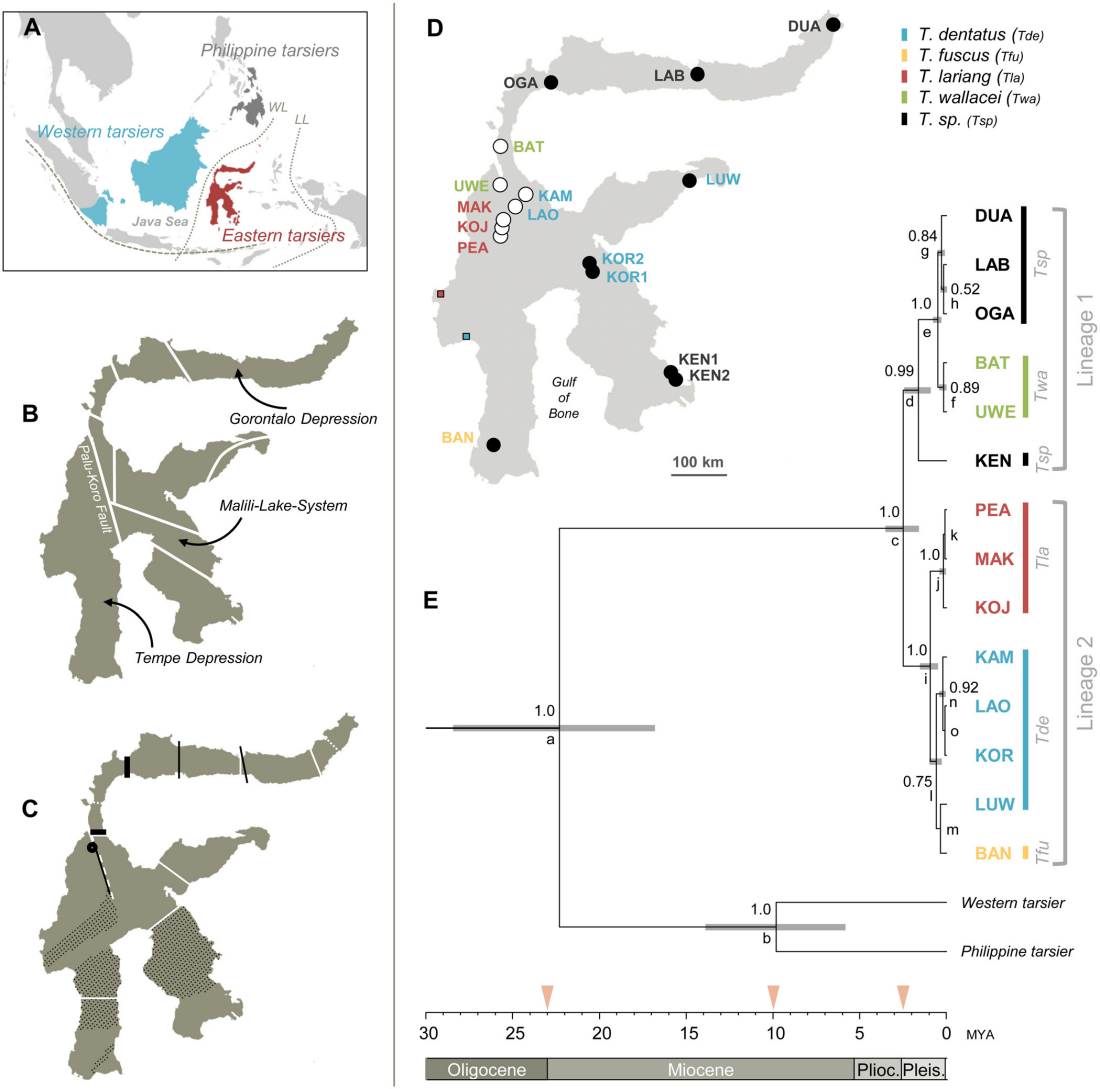
Informal maps of Sulawesi and tarsier phylogenetic trees. A) Malay Archipelago and distribution of extant tarsiers. Dotted lines: Western (Wallace Line, WL) and eastern boundary (Lydekker Line, LL) of the Wallacea region. Dashed line: Sunda Arc. B) Main tectonic sutures on Sulawesi [1]. Arrows point to topographically significant regions. C) Species ranges of distinct lineages. White continuous lines: Macaque and toad hybrid zones, white dotted lines: Toad ranges deviating from the nearest macaque hybrid zone [4]; Continuous black lines: Distribution of tarsier acoustic forms [9, 17], thick black lines and circle indicate the discontinuous range of *T*. *wallacei* [17], in black dotted areas, tarsier species boundaries are yet to be determined; White dashed line: population differentiation in a Sulawesian bat species, *Thoopterus nigrescens* [5]. D) Study sites on Sulawesi indicated by white dots (2001–2008; BAT: Batusuya; KAM: Kamarora; KOJ: Koja; LAO: Laone; MAK: Make; PEA: Peana; UWE: Uwemanje), black dots (2009–2010; BAN: Bantimurung; DUA: Duasaudara; KEN: Kendari; KOR: Korosule; LAB: Labanu; LUW: Luwuk; OGA: Ogatemuku) and coloured squares. Squares mark sites where only tarsier vocalizations were recorded (blue: *T*. *dentatus*-like, red: *T*. *lariang*-like). Colored labels mark populations with taxonomic affiliation (see color key at the top right). E) Time-calibrated multilocus Bayesian species tree with posterior probabilities (pp) above 0.5 for internal nodes and 95% confidence intervals on divergence time estimates indicated by grey node bars. Lower case letters correspond to node names in Table 2. Arrows at the time-scale point to the Sundaland/Sula-Spur collision at 23 MYA [1] and significant sea-level lowstands at 10 and 2.5 MYA [19].

Situated at the triple junction of the Australian, Eurasian, and Pacific plates the Indonesian island of Sulawesi is part of the tectonically most active region in the world. Geologically, Sulawesi has been formed by converging micro-terranes that successively emerged due to subduction zone processes since the Miocene and fused into one another not before the early Pliocene period around 5 MYA [1]. Today, the island covers more than half of the Wallacean terrestrial biosphere (Fig 1A), and therefore it reflects the hotspot of intermingling Asian and Australian biota [2]. Sulawesi hosts a rich and highly endemic fauna with overlapping geographic ranges between disparate taxa [3–8]. It is not surprising that this pattern of regional endemism has been associated with the complex geological evolution of Sulawesi, where Neogene and Quaternary tectonic and climatic changes are assumed to be the key factors driving radiations/divergence [4, 7]. Apart from the central mountains several fault systems and basins are prominent relief features on Sulawesi [1, 2] (Fig 1B). At least some of these physiographic landmarks determine geographic borders of currently known endemism areas [4, 5, 7, 9] (Fig 1C). It is thus conceivable that each region flanked by these suture lines and depressions temporarily formed its own biogeographic entity, likely isolated from each other through water influx during interglacial periods. However, phylogeographic patterns also depend on time and location of colonization and subsequent range expansion [4]. Likewise, lineage-specific habitat preferences, dispersal abilities and sex-biased dispersal behavior can affect population structuring [4, 5, 10–13], thus leading to phylogenetic signals not matching major geological events. Therefore it remains challenging to subdivide Sulawesi into general biotic units, both for conservation purposes [4] and for the generation of testable biogeographic hypotheses [14].

We here elucidate colonization and diversification of a group of widely distributed arboreal island endemics, the Sulawesi tarsier, whose phylogeography is thought to reflect some very general phenomena related to the island´s biogeography. Modern tarsiers represent the oldest lineage of extant haplorhine primates [15]. Their geographical range is restricted to insular Southeast Asia, where they fall into the three spatially and evolutionary distinct clades of Western (*Tarsius bancanus*), Philippine (*Tarsius syrichta*), and Sulawesi tarsiers (Fig 1A). The latter group inhabiting Sulawesi and smaller surrounding islands is the most speciose tarsier clade with *Tarsius dentatus*, *T*. *fuscus*, *T*. *lariang*, *T*. *pumilus*, and *T*. *wallacei* being endemic to mainland Sulawesi [9, 16]. Further, several other yet unclassified Sulawesi tarsier taxa have been recognized [9]. Those being part of this study are referred to as *Tarsius sp*. and/or termed according to their respective sample site. Sulawesi tarsiers show a high degree of regional endemism reported on the level of both genetic makeup and vocalization [7, 9]. However, neither phenotypic nor molecular data (autosomal STR genotypes, Y chromosomal and mitochondrial haplotypes) sampled so far allowed a thorough and comprehensive inference of a pan-Sulawesi tarsier phylogeography, either because studies were focused on Central-Sulawesian populations [7, 17], or because the applied molecular marker was not adequate for resolving phylogenetic relationships among closely related taxa [9]. Therefore, we analyzed the sex-determining region of the Y-chromosome (SRY) and a set of five autosomal and taxonomic widely applicable phylogenetic markers (ABCA1, ADORA3, AXIN1, RAG1, and TTR) [18] in a tarsier sampling with a broad geographic coverage reflecting both geological and vocal features (Fig 1B–1D and Table 1). On this basis we inferred phylogenetic relationships of distinct tarsier populations and, furthermore, tested the plausibility of tectonic and climatic forces driving speciation on Sulawesi. To this end we correlate paleo-environmental and paleo-geographic data with divergence times among Sulawesi tarsiers. In addition we asked whether and to what extent range fragmentation or lineage-specific life history traits shaped current phylogeographic patterns of these group-living nocturnal primates comparing the tarsier´s spatial distribution to that of other island endemics.

**Table 1:** Overview of individuals analyzed for five autosomal loci, the Cytb gene, and the SRY gene. x: New sequences are printed in bold type, sequences obtained from previous studies appear in normal lettering.

#	Individual	Species	Population	Cytb	SRY	ABCA1	ADORA3	AXIN1	RAG1	TTR
1	CD01	*Tarsius sp*.	OGA	**x**						
2	CD02	*Tarsius sp*.	OGA	**x**		**x**	**x**	**x**	**x**	**x**
3	CD03	*Tarsius sp*.	OGA	**x**						
4	CD04	*Tarsius sp*.	OGA	**x**	**x**	**x**	**x**	**x**	**x**	**x**
5	CD05	*Tarsius sp*.	OGA	**x**	**x**					
6	CD06	*Tarsius sp*.	OGA	**x**						
7	CD07	*Tarsius sp*.	OGA	**x**						
8	CD08	*Tarsius sp*.	OGA	**x**						
9	CD09	*Tarsius sp*.	OGA	**x**	**x**					
10	CD10	*Tarsius sp*.	OGA	**x**						
11	CD11	*Tarsius sp*.	OGA	**x**	**x**					
12	CD12	*Tarsius sp*.	OGA	**x**	**x**					
13	CD13	*Tarsius dentatus*	KOR1	**x**		**x**	**x**	**x**	**x**	**x**
14	CD14	*Tarsius dentatus*	KOR1	**x**	**x**					
15	CD15	*Tarsius dentatus*	KOR2	**x**						
16	CD16	*Tarsius dentatus*	KOR2	**x**	**x**	**x**	**x**	**x**	**x**	**x**
17	CD17	*Tarsius dentatus*	KOR2	**x**						
18	CD18	*Tarsius dentatus*	KOR2	**x**						
19	CD19	*Tarsius dentatus*	LUW	**x**	**x**	**x**	**x**	**x**	**x**	**x**
20	CD20	*Tarsius dentatus*	LUW	**x**						
21	CD21	*Tarsius dentatus*	LUW	**x**						
22	CD22	*Tarsius dentatus*	LUW	**x**						
23	CD23	*Tarsius dentatus*	LUW	**x**	**x**					
24	CD24	*Tarsius dentatus*	LUW	**x**		**x**	**x**	**x**	**x**	**x**
25	CD25	*Tarsius dentatus*	LUW	**x**	**x**					
26	CD26	*Tarsius dentatus*	LUW	**x**	**x**					
27	CD27	*Tarsius dentatus*	LUW	**x**						
28	CD28	*Tarsius dentatus*	LUW	**x**	**x**					
29	CD29	*Tarsius sp*.	LAB	**x**						
30	CD30	*Tarsius sp*.	LAB	**x**	**x**					
31	CD31	*Tarsius sp*.	LAB	**x**						
32	CD32	*Tarsius sp*.	LAB	**x**						
33	CD33	*Tarsius sp*.	LAB	**x**	**x**	**x**	**x**	**x**	**x**	**x**
34	CD34	*Tarsius sp*.	LAB	**x**		**x**	**x**	**x**	**x**	**x**
35	CD35	*Tarsius sp*.	LAB	**x**						
36	CD36	*Tarsius sp*.	LAB	**x**						
37	CD37	*Tarsius sp*.	LAB	**x**	**x**					
38	CD38	*Tarsius sp*	LAB	**x**						
39	CD39	*Tarsius sp*	KAN1	**x**						
40	CD40	*Tarsius sp*.	KEN1	**x**		**x**	**x**	**x**	**x**	**x**
41	CD41	*Tarsius sp*.	KEN2	**x**	**x**	**x**	**x**	**x**	**x**	**x**
42	CD41	*Tarsius sp*	KEN2	**x**						
43	CD43	*Tarsius sp*	KEN2	**x**						
44	CD44	*Tarsius sp*.	DUA	**x**		**x**	**x**	**x**	**x**	**x**
45	CD45	*Tarsius sp*.	DUA	**x**	**x**					
46	CD46	*Tarsius sp*.	DUA	**x**	**x**	**x**	**x**	**x**	**x**	**x**
47	CD47	*Tarsius sp*.	DUA	**x**						
48	CD48	*Tarsius sp*.	DUA	**x**	**x**					
49	CD49	*Tarsius sp*.	DUA	**x**						
50	CD50	*Tarsius sp*.	DUA	**x**						
51	CD51	*Tarsius sp*.	DUA	**x**	**x**					
52	CD52	*Tarsius sp*.	DUA	**x**	**x**					
53	CD53	*Tarsius sp*.	DUA	**x**						
54	CD54	*Tarsius sp*.	DUA	**x**						
55	CD55	*Tarsius sp*.	DUA	**x**						
56	CD56	*Tarsius fuscus*	BAN	**x**						
57	CD57	*Tarsius fuscus*	BAN	**x**	**x**					
58	CD58	*Tarsius fuscus*	BAN	**x**						
59	CD59	*Tarsius fuscus*	BAN	**x**						
60	CD60	*Tarsius fuscus*	BAN	**x**	**x**	**x**	**x**	**x**	**x**	**x**
61	CD61	*Tarsius fuscus*	BAN	**x**						
62	CD62	*Tarsius fuscus*	BAN	**x**		**x**	**x**	**x**	**x**	**x**
63	CD63	*Tarsius fuscus*	BAN	**x**						
64	CD64	*Tarsius fuscus*	BAN	**x**	**x**					
65	CD65	*Tarsius fuscus*	BAN	**x**						
66	K02	*Tarsius dentatus*	KAM	x		**x**	**x**	**x**	**x**	**x**
67	K03	*Tarsius dentatus*	KAM	x						
68	K04	*Tarsius dentatus*	KAM	x		**x**	**x**	**x**	**x**	**x**
69	K05	*Tarsius dentatus*	KAM	x						
70	K06	*Tarsius dentatus*	KAM	x	x					
71	K07	*Tarsius dentatus*	KAM	x						
72	K11	*Tarsius dentatus*	KAM	x						
73	K12	*Tarsius dentatus*	KAM	x						
74	K15	*Tarsius dentatus*	KAM	x						
75	K16	*Tarsius dentatus*	KAM	x						
76	K17	*Tarsius dentatus*	KAM	x						
77	K18	*Tarsius dentatus*	KAM	x						
78	K19	*Tarsius dentatus*	KAM	x						
79	K20	*Tarsius dentatus*	KAM	x	x					
80	K21	*Tarsius dentatus*	KAM	x	x					
81	K22	*Tarsius dentatus*	KAM	x						
82	K24	*Tarsius dentatus*	KAM	x						
83	K27	*Tarsius dentatus*	KAM	x						
84	K28	*Tarsius dentatus*	KAM	x						
85	K29	*Tarsius dentatus*	KAM	x						
86	K30	*Tarsius dentatus*	KAM	x						
87	K31	*Tarsius dentatus*	KAM	x	x					
88	K32	*Tarsius dentatus*	KAM	x						
89	T06	*Tarsius lariang*	MAK	x	x					
90	T07	*Tarsius lariang*	MAK	x	x					
91	T08	*Tarsius lariang*	MAK	x		**x**	**x**	**x**	**x**	**x**
92	T09	*Tarsius lariang*	MAK	x	x	**x**	**x**	**x**	**x**	**x**
93	T10	*Tarsius lariang*	MAK	x	x					
94	T11	*Tarsius lariang*	MAK	x						
95	T12	*Tarsius lariang*	MAK	x	x					
96	T15	*Tarsius lariang*	PEA	x						
97	T16	*Tarsius lariang*	PEA	x	x					
98	T17	*Tarsius lariang*	PEA	x	x					
99	T18	*Tarsius lariang*	PEA	x	x					
100	T19	*Tarsius lariang*	PEA	x						
101	T20	*Tarsius lariang*	PEA	x						
102	T21	*Tarsius lariang*	PEA	x	x					
103	T22	*Tarsius lariang*	PEA	x						
104	T23	*Tarsius lariang*	PEA	x						
104	T24	*Tarsius lariang*	PEA	x	x	**x**	**x**	**x**	**x**	**x**
106	T25	*Tarsius lariang*	PEA	x	x					
107	T26	*Tarsius lariang*	PEA	x						
108	T27	*Tarsius lariang*	PEA	x						
109	T28	*Tarsius lariang*	PEA	x						
110	T29	*Tarsius lariang*	PEA	x	x					
111	T30	*Tarsius lariang*	PEA	x						
112	T31	*Tarsius lariang*	PEA	x	x					
113	T32	*Tarsius lariang*	PEA	x	x					
114	T33	*Tarsius lariang*	PEA	x						
115	T34	*Tarsius lariang*	PEA	x	x					
116	T35	*Tarsius lariang*	PEA	x						
117	T36	*Tarsius lariang*	PEA	x						
118	T37	*Tarsius lariang*	PEA	x						
119	T38	*Tarsius lariang*	PEA	x	x					
120	T39	*Tarsius lariang*	PEA	x		**x**	**x**	**x**	**x**	**x**
121	T40	*Tarsius lariang*	PEA	x						
122	T41	*Tarsius lariang*	PEA	x						
123	T42	*Tarsius lariang*	PEA	x						
124	T43	*Tarsius lariang*	KOJ	x						
125	T44	*Tarsius lariang*	KOJ	x						
126	T45	*Tarsius lariang*	KOJ	x						
127	T46	*Tarsius lariang*	KOJ	x	x	**x**	**x**	**x**	**x**	**x**
128	T47	*Tarsius lariang*	KOJ	x		**x**	**x**	**x**	**x**	**x**
129	T105	*Tarsius dentatus*	LAO	x	x					
130	T106	*Tarsius dentatus*	LAO	x	x					
131	T107	*Tarsius dentatus*	LAO	x	x					
132	T108	*Tarsius dentatus*	LAO	x	x					
133	T109	*Tarsius dentatus*	LAO	x	x					
134	T110	*Tarsius dentatus*	LAO	x						
135	T111	*Tarsius dentatus*	LAO	x	x	**x**	**x**	**x**	**x**	**x**
136	T112	*Tarsius dentatus*	LAO	x	x	**x**	**x**	**x**	**x**	**x**
137	SM24	*Tarsius wallacei*	BAT	x						
138	SM25	*Tarsius wallacei*	BAT	x						
139	SM26	*Tarsius wallacei*	BAT	x	x	**x**	**x**	**x**	**x**	**x**
140	SM27	*Tarsius wallacei*	BAT	x						
141	SM28	*Tarsius wallacei*	BAT	x		**x**	**x**	**x**	**x**	**x**
142	SM29	*Tarsius wallacei*	BAT	x						
143	SM30	*Tarsius wallacei*	BAT	x	x					
144	SM31	*Tarsius wallacei*	BAT	x						
145	SM32	*Tarsius wallacei*	BAT	x						
146	SM33	*Tarsius wallacei*	UWE	x	x	**x**	**x**	**x**	**x**	**x**
147	SM34	*Tarsius wallacei*	UWE	x	x					
148	SM35	*Tarsius wallacei*	UWE	x	x	**x**	**x**	**x**	**x**	**x**
149	SM36	*Tarsius wallacei*	UWE	x						
150	SM37	*Tarsius wallacei*	UWE	x	x					
151	SM38	*Tarsius wallacei*	UWE	x	x					
152	TSY	*Tarsius syrichta*	N/A	x	**x**	**x**	**x**	**x**	**x**	**x**
153	TBA	*Tarsius bancanus*	N/A	x		**x**	**x**	**x**	**x**	**x**

## Material and Methods

### Sample collection and preparation

We collected tissue samples of 65 tarsiers at nine sites in seven study areas located throughout Sulawesi between 2009 and 2010 (Fig 1D, blacks dots). Tarsiers were localized by tracing their scent marks and morning duet calls to their sleeping site. For collecting DNA samples, tarsiers were captured by mist-netting near that site at dusk and dawn. Small ear biopsies were taken from the tip of the pinna and stored in Urea-EDTA buffer [6 M Urea, 10 mM Tris/HCl (pH 8), 10 mM EDTA, 125 mM NaCl and 1% SDS]. To avoid injuring small blood vessels in the translucent pinna of the ear, we checked their course with the help of a flashlight. We then obtained small ear biopsies (triangles with 2 mm side lengths) from the thin tip of the pinna using scissors and tweezers (cleaned with water, ethanol, and wipes) and applied iodine solution to the tarsier ear. Animals did not required sedation prior to the biopsy. All tarsiers were subsequently released alive at their respective capture site. Permits to capture and sample wild tarsiers (no. SK. 198/IV-SET/2009 and SK. 50/IV-SET/2010), and to enter protected areas (no. SI. 86/Set-3/2009, SI 17/Set-3/2010, and SI. 025/BTNBABUL-1/PK/2010) were issued by the Indonesian CITES management authority, the Directorate General of Forest Protection and Nature Conservation (PHKA). We extracted DNA from tissue samples using a DNeasy Blood and Tissue Kit (Qiagen). All specimens were subjected to whole genome amplification (WGA) using the GenomiPhi DNA Amplification Kit (GE Healthcare) to increase the amount of limited genomic material and to meet national and CITES export and import requirements in terms of CITES Appendix-II species. CITES permits covered export (no. 0517/IV/SATS-LN/2010) and import (E-0179/10) of WGA samples from Indonesia to Germany.

Additionally, our study comprised WGA-amplified DNA probes of 95 central Sulawesi tarsiers from seven populations (Fig 1D) sampled during previous studies [7, 17]. DNA samples of the Philippine and of the Western tarsier were provided by J. Brosius and J. Schmitz (University of Muenster, Germany), and by Y. Rumpler (Les Hôpitaux Universitaires de Strasbourg, France), respectively.

### Sequence data collection

In our analyses we included five nuclear autosomal loci (exonic: ADORA3, AXIN1, RAG1; intronic: ABCA1, TTR) of the “Phylogenomic toolkit” [18], in total yielding around 3400 bp of sequence information. Primers and conditions were partly modified (S1 Table) and applied to a pruned sample set comprising 28 specimens from Sulawesi, and one specimen from each the Philippine and the Western tarsier (Table 1, GenBank accession numbers KP642169-KP642408 and KP642434-KP642493) for PCR amplification and sequencing (for more details see S1 Text). Sulawesian populations/study areas were represented by two individuals each constituting a terminal taxon of a preliminary Cytochrome b maximum likelihood tree estimated from sequence data of 151 specimens (Table 1, S2 Text and S1 Fig). Mitochondrial sequences were newly amplified for the 65 individuals sampled in 2009 and 2010 (Table 1, GenBank accession numbers KR337026-KR337090) and obtained from previous studies [7, 17, 20] (Table 1 and S2 Text).

We further PCR amplified and sequenced (for more details see S1 Text) a fragment of the Y-chromosomal SRY gene for 24 males (630 bp, Genbank accession numbers KPKP642409-KP642433, for more details see Table 1 and S1 Text) adopting PCR primers and conditions from Merker et al. [7]. Data sets from former studies and comprising 35 males [7, 17] (Table 1, Genbank accession numbers FJ614510-13, FJ614517-21, FJ614523-34, FJ614562-68, HM115985-91) were incorporated for further analyses.

Sequencing was performed on an ABI 3130xl genetic analyzer (Applied Biosystems). Sequences were edited in BioEdit 7.0.9.0 [21] and aligned using Muscle v. 3.8.31 [22]. Multiple sequence alignments were generated using Gblocks v. 0.91b [23] and refined manually.

### Sequence data analyses

Models of DNA sequence evolution were selected based on Akaike´s information criterion corrected for small sample sizes (AICc) using Treefinder v. March 2011 [24]. We included substitution models for each locus (S2 Table) into subsequent phylogenetic analyses as outlined below.

Multilocus species trees were estimated from combined nuclear markers of the “Phylogenomic Toolkit” [18] employing *Beast v. 1.6.2 [25]. We consulted the ucld.stdev and the coefficient of variation parameter of preliminary simulations under an uncorrelated relaxed lognormal clock to reveal deviations from a global clock. Both parameters indicated low rate variation among lineages Thus, we used a strict molecular clock model to analyze multilocus sequence data. We ran ten independent chains of 1x10E7 length sampling every 10,000 generations and leaving the other priors at their default values. For MCMC diagnostics we examined ESS values of the Beast output in Tracer v. 1.5 [26] and Awty [27]. In all analyses, ESS values were generally above 1,000, chains converged and mixed well. Awty´s plots for comparisons of split frequencies across all paired MCMC chains further confirmed convergence. We combined two of the ten analyses to create a final multilocus species tree.

We reconstructed phylogenies for Y-chromosomal data conducting maximum likelihood (ML) and Bayesian analyses. ML trees were estimated using Garli v. 2.0 [28] with two search replicates and stepwise-addition starting trees. Bootstrap support was assessed by 100 replicates of which a majority rule consensus tree was inferred with the program Consense of the Phylip package v. 3.69 [29, 30]. Bayesian phylogenies were generated by MrBayes v. 3.2 [31]. We ran four independent analyses of 5x10E6 with four Markov chains, sampling every 1,000 generations. We verified convergence of the four runs by examining the average standard deviation of split frequencies (ASDSF), the stationarity of log likelihood values (LnL) of the cold chain, the chain swap acceptance rate, and the potential scale reduction factor (PSRF). ASDSF decreased to ≤ 0.01 within the first 800,000 generations, LnL reached stationarity distribution in the first thousand generations, the swap acceptance rate between chains was between 20–60%, and PSRF values were close or equal to 1. On this basis, we concluded that all four runs converged, discarded the burn-in (25%) and built a majority rule tree from the resulting trees.

Moreover, we reconstructed haplotype networks of each autosomal marker and of the SRY gene based on non-identical sequences and using the TCS method [32] as implemented in the PopART package v. 1.7 [33].

### Estimating divergence times

We also applied *Beast v. 1.6.2 on the five combined nuclear gene markers for dating divergence within *Tarsius*. Divergence time estimates recently published by Perelman et al. [34] were used to calibrate seven nodes outside the genus *Tarsius*. For this we complemented our multiple sequence alignments with anthropoid and strepsirhine species (sequence resources are listed in S3 Table) and created eight taxon sets: Homininae, Hominidae, Hominoidea, Catarrhini, Anthropoidea, *Tarsius*, Haplorhini (monophyletic), and Strepsirrhini. Models of nucleotide evolution (S2 Table) and substitution rates were determined as described above. Accordingly, divergence times (Table 2 and S4 Table) were estimated under an uncorrelated lognormal relaxed clock using a gamma distribution as prior on branch-specific substitution rates (shape 0.001, scale 1000). We applied normal distribution priors to the seven calibration points and added information for the mean time to the most recent common ancestor (tmrca) in million years ago (MYA) and a standard deviation, both derived from node ages and 95% credibility intervals estimated by Perelman et al. [34]. For node calibration we set divergence times as follows: 1) Homininae 6.68/0.64, 2) Hominidae 18.07/0.81, 3) Catarrhini 31.77/3.06, 4) Anthropoidea 43.46/2.45, 5) Haplorhini 82.16/6.83, 6) Strepsirrhini 67.69/4.46, 7) Primates 87.27/5.69. Three independent Beast analyses were run for 5x10E7 generations saving every 10,000th tree. Trace files were analyzed in Tracer v. 1.5 [26] and Awty [27] to confirm convergence of the Markov chain.

**Table 2:** Divergence time estimates and node support.

		Median	95% HPD*		
Node	Node label	Node age (MYA)	lower	upper	pp**
*Tarsius*	a	22.31	16.81	28.43	1.00
	b	9.82	5.84	13.90	1.00
*Lineage 1*	c	2.50	1.60	3.54	1.00
	d	1.63	0.92	2.45	0.99
	e	0.51	0.29	0.78	1.00
	f	0.18	0.00	0.43	0.89
	g	0.30	0.10	0.54	0.84
	h	0.18	0.00	0.38	0.52
*Lineage 2*	i	0.95	0.50	1.53	1.00
	j	0.20	0.03	0.42	1.00
	k	0.10	0.00	0.27	0.41
	l	0.59	0.28	0.98	0.75
	m	0.35	0.05	0.73	0.41
	n	0.22	0.05	0.43	0.92
	o	0.10	0.00	0.28	0.40

Alphabetical ordering of nodes corresponds to alphabetically labeled tree nodes in Fig 1E.

* Lower and upper bound of the highest posterior density

** Posterior probability

## Results

### Nuclear DNA Sequence Data

**Fig 2:**
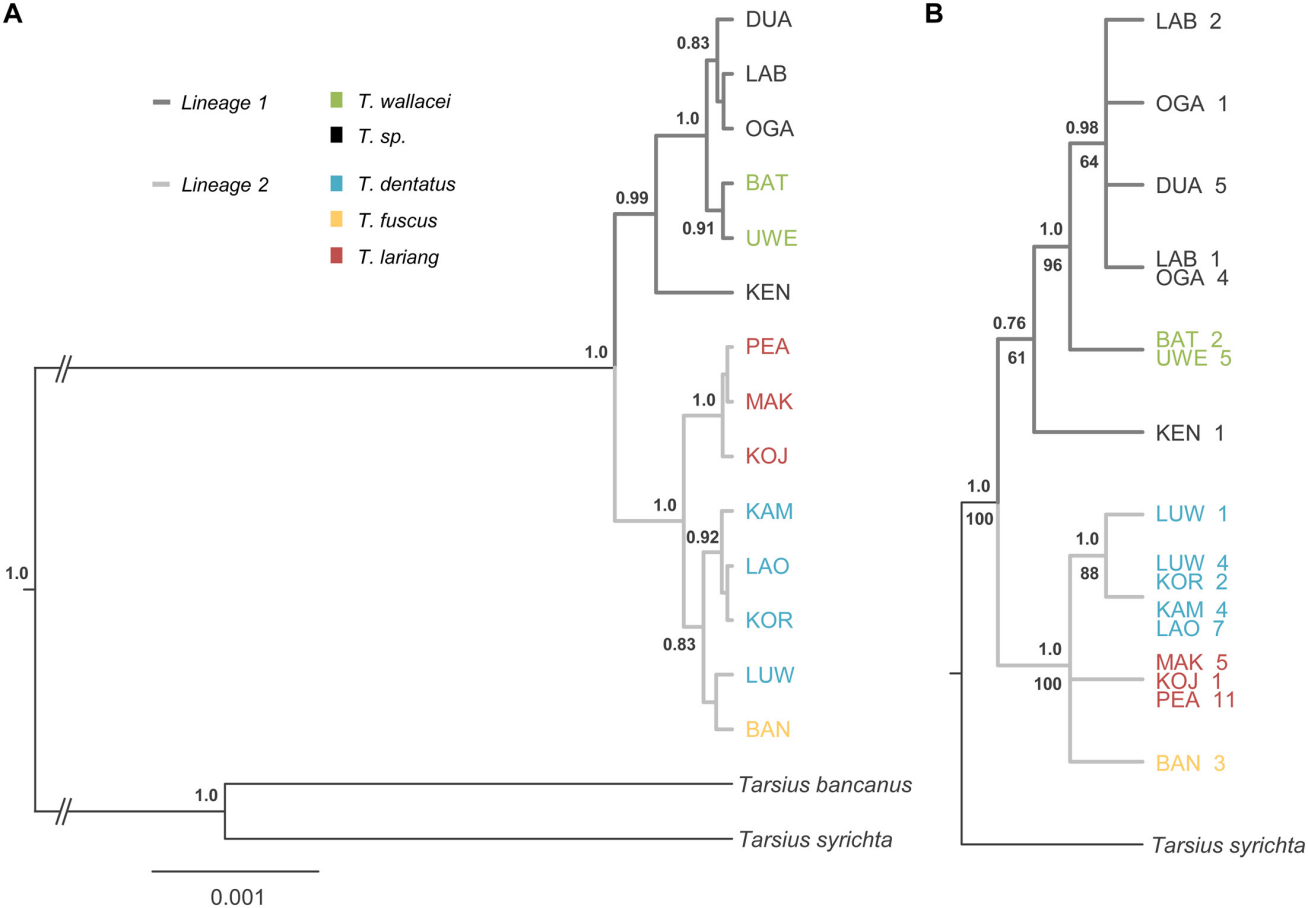
Species- and gene tree. A) Multilocus Bayesian species tree. Numbers at nodes indicate posterior probability values above 0.5. B) Genealogy of ten SRY-haplotypes from 59 males. Node support values of Bayesian/maximum likelihood analyses above/below nodes represent posterior probabilities (pp) and bootstrap values (%), respectively. Numbers behind the population label/sampling site correspond to the number of males carrying the respective haplotype. Each terminal branch represents a distinct SRY haplotype.

Multilocus Bayesian species tree inference revealed two evolutionary lineages (pp = 1.0) on Sulawesi (Fig 2A) hereafter referred to as lineage 1 and lineage 2 (Figs 2A and 1E). The southeastern population of KEN represents the most basal group within lineage 1 and is placed as a sister group to all northern populations (pp = 0.99). Among those, the north-central *Tarsius wallacei* (BAT, UWE) is distinct from the northern DUA, LAB, and OGA populations and forms a well-supported monophyletic group (pp = 1.0). Within lineage 2, the west-central *T*. *lariang* (PEA, MAK, KOJ) is sister group to *T*. *fuscus* (BAN) and *T*. *dentatus*, (pp = 1.0). KOR was phylogenetically allied with *T*. *dentatus* (pp = 0.92), while the placement of LUW as sister species to BAN gained only weak support (pp = 0.42). Furthermore, in species tree analysis Sulawesi tarsiers were recovered as monophyletic with respect to Western and Philippine tarsiers (pp = 1.0), without having guided tree search by supplying topological constraints on outgroup nodes. Statistical parsimony networks of autosomal alleles (S2–S6 Figs) tend in general to confirm the results of the multilocus species tree inference. In ABCA1-, AXIN1, and RAG1-networks (S2, S4 and S5 Figs) alleles of lineage 1-populations form a continuous cluster. Alleles of KEN are most basal within these clusters. The sharing of alleles was higher between OGA, LAB, and DUA than between these populations, KEN and *T*. *wallacei*, perhaps being indicative of their more recent divergence. For the same three loci alleles of *T*. *dentatus*, *T*. *fuscus* and *T*. *lariang* define species- and lineage-specific clusters pointing to a recent common ancestor. In ADORA3- and TTR-networks (S3 and S6 Figs) species-clusters only fairly correspond to those inferred from the multilocus species tree analysis. The comparatively short lengths of ADORA3-sequences could be responsible for the low information value regarding phylogenetic relationships. Masking gap positions (see PopART documentation) led to the collapsing of several alleles into one node and, therefore, may also to the loss of relevant phylogenetic information in the TTR-network. Overall, parsimony networks of autosomal alleles show that, despite patterns of incomplete lineage sorting, speciation processes in Sulawesi tarsiers are well advanced.

**Fig 3:**
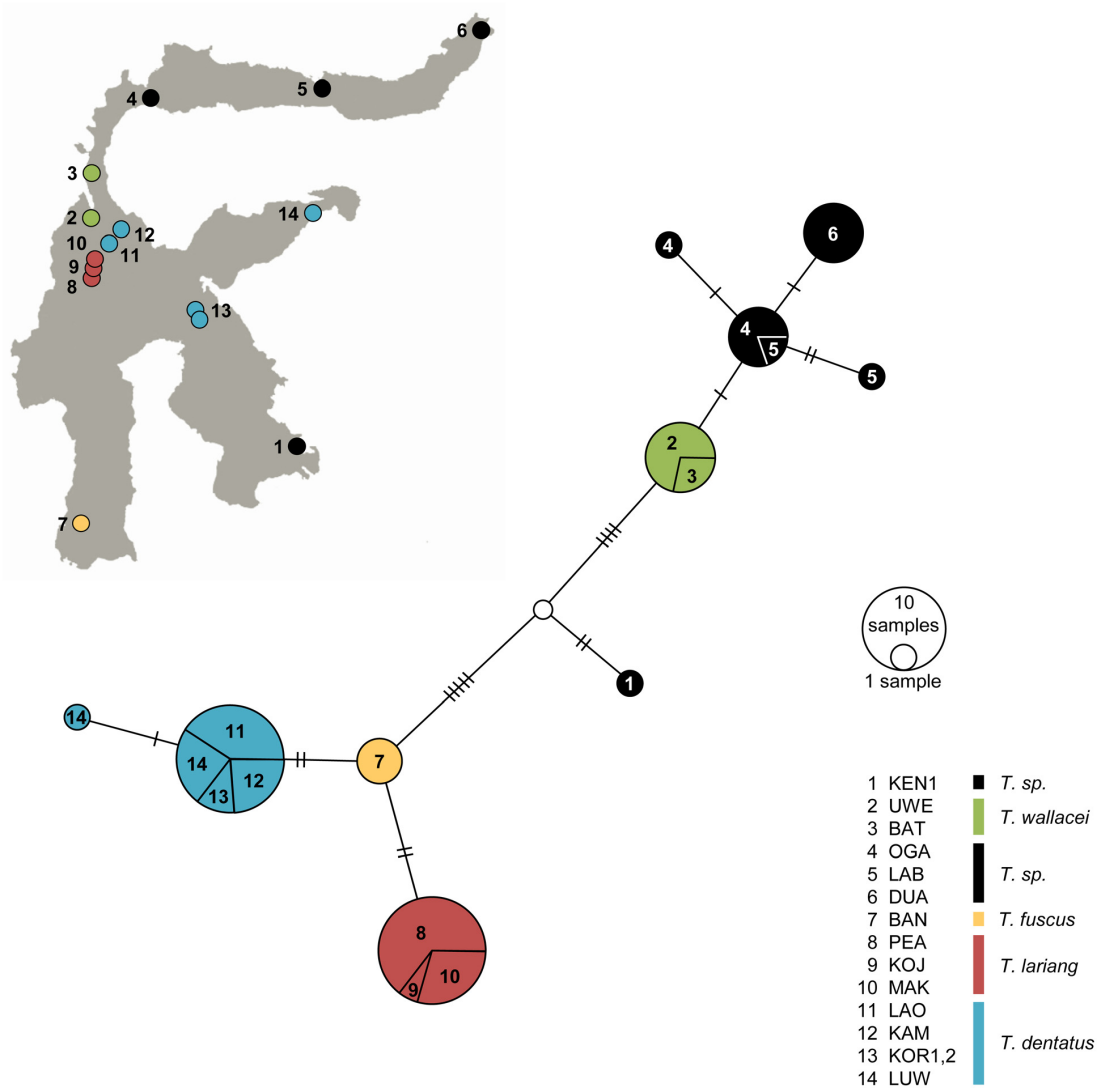
SRY haplotype network. Shown are the statistical parsimony network based on 10 SRY-haplotypes obtained from 59 males and haplotype distribution on Sulawesi. The white circle indicates an inferred missing haplotype. Mutation steps are shown as hatch marks.

The pan-Sulawesi sample comprised ten SRY-haplotypes found in 59 males. Six haplotypes were unique to their sample location (BAN, DUA, KEN, LAB, LUW, OGA), four haplotypes were species-specific and/or shared between adjacent populations (Figs 1D and 2B). We reconstructed ML and Bayesian gene trees based on one Philippine and ten Sulawesi SRY haplotypes (Fig 2B). Phylogenetic analyses revealed that Sulawesi tarsiers split into two major paternal lineages (ML bootstrap value = 100, pp = 1.0). As inferred by Bayesian species tree inference KEN was grouped together with *T*. *wallacei*, DUA, LAB, and OGA. *T*. *fuscus*, *T*. *lariang*, *T*. *dentatus*, KOR, and LUW constituted the second lineage. Within lineage 2 *T*. *dentatus* clustered with KOR and LUW, with one haplotype being common to all six study sites and another haplotype being unique to LUW (Fig 2B). The haplotype network (Fig 3) broadly confirms these results by separating northern populations of lineage 1 from lineage 2-species and by the slightly lower sequence divergence between the KEN-haplotype and haplotype-clusters of northern populations. Within lineage 2 all three species (*T*. *dentatus*, *T*. *fuscus*, and T. *lariang*) form contiguous clusters.

### Divergence Times

Tarsiers have a scarce fossil record [35, 36] notoriously impeding the choice of calibration points for a robust estimation of divergence times on the tarsier lineage. We therefore used mean node age estimates of a recently published primate phylogeny based on comprehensive genomic and fossil data [34]. Furthermore, we applied a relaxed clock method to increase the accuracy of divergence time estimation [37].

For dating speciation events within *Tarsius* (Table 2) we added prior densities to ancestral nodes of anthropoid and strepsirrhine taxonomic groups using calibration information outlined in Perelman et al. [34]. Accordingly, the Eastern lineage split from other crown tarsiers between late Oligocene and early Miocene (median node age calibration: 22.3 MYA, 95% confidence intervals ranging from 16.8–28.4 MYA, Fig 1E). Crown Sulawesi tarsiers further diversified into two major lineages not before Plio-Pleistocene (median node age calibration: 2.5 MYA, 95% confidence intervals ranging from 1.6–3.5 MYA, Fig 1E). Both lineages underwent speciation during the Pleistocene. Divergence between *T*. *bancanus* and *T*. *syrichta* was dated to about 10 MYA (median node ages: 9.8 MYA, 95% confidence interval: 5.8–13.9 MYA; pp = 1.0).

## Discussion

### Tarsier Phylogeography and Island Formation

**Fig 4:**
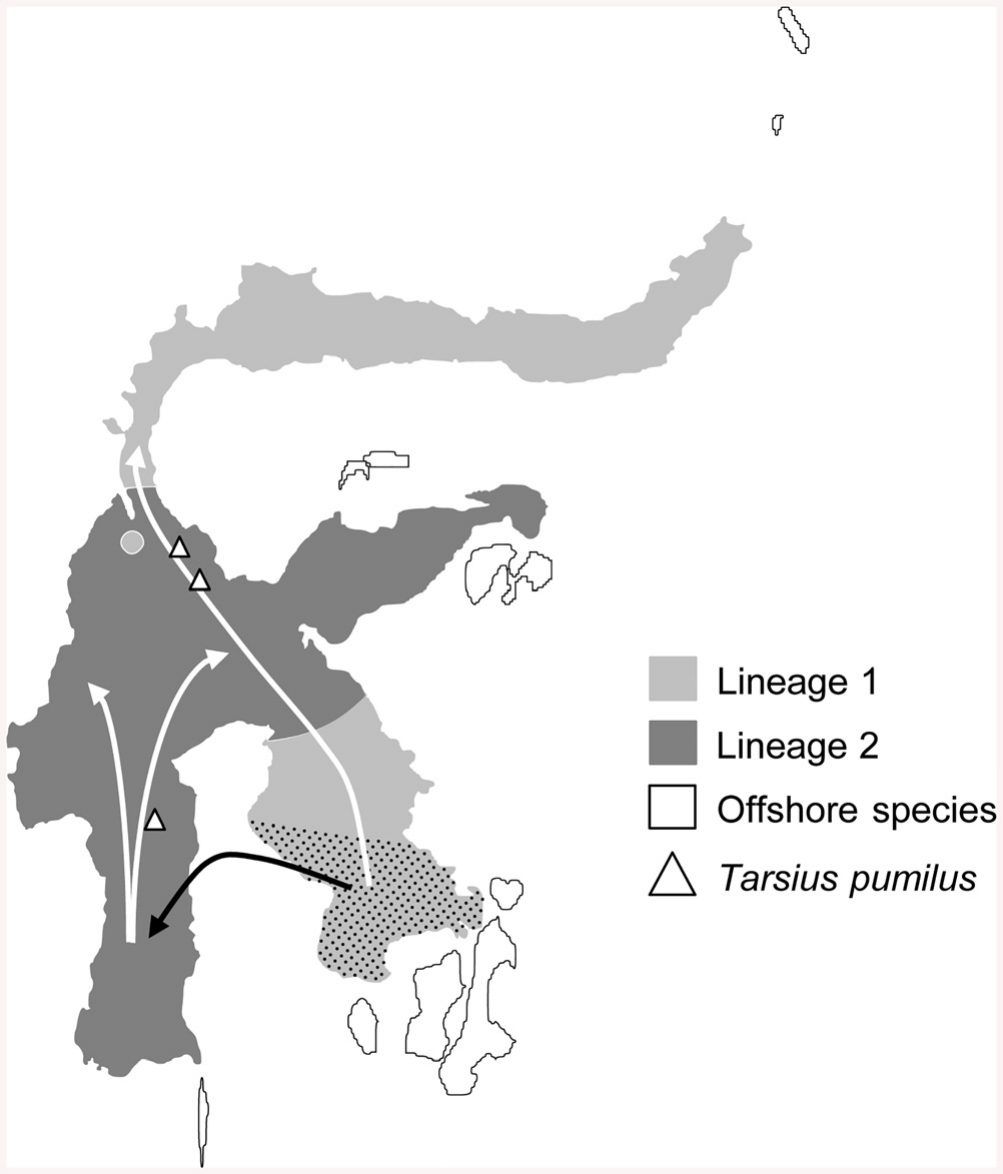
Waves of dispersal and geographical distribution of Sulawesi tarsiers. White and black arrows symbolize our proposed dispersal routes of the two lowland lineages on Sulawesi. The dotted pattern indicates a possible origin of the extant Sulawesi tarsier population that is unknown so far.

Over the past two decades paleo-geographic maps of the Wallacea region have been continually revised and adapted to the current state of knowledge [1, 38–42]. Reconstructing areas of subaerial land from the absence of marine deposits is still a geologists’ method of choice. The call for cross-fertilization of geological and biological inferences, however, became louder over the last years [40]. Thus, tracing the dispersal history of Sulawesi’s old endemics might add new tesserae to reconstruct the geological and environmental setting of past epochs. Arboreal tarsiers and their extinct relatives occupied tropical rainforests in Southeast Asia since the Eocene epoch [35]. They are strong habitat specialists, for which reason the deduction of their dispersal routes may be informative about forested areas at times of dispersal. In order to identify drivers of faunal diversification, we interpret estimated divergence times among extant tarsiers in relation to past geological and paleoclimatic events. We further linked our divergence time estimates to proposed land and sea distributions at the paleo-Sulawesi archipelago [40–43] and to present-day topographical maps indicating interglacial shallow seas and presumably flooded low lying land areas at times of historical sea level increase. We used this information to locate potential land bridges and contemporary reproductive barriers in the geological past and thus inferred the most-parsimonious albeit hypothetical dispersal pattern resulting in modern species distribution (Fig 4).

Our genetic analyses do not contradict a monophyletic origin of Sulawesi tarsiers and point to their separation from other crown tarsiers (also including the last common ancestor of Philippine and Western tarsiers) between late Oligocene and early Miocene (Fig 1E). This period was marked by a notable drop in sea level [19] and might therefore have facilitated the colonization of Wallacea via dispersal [43]. Moreover, the estimated median node age at 22.3 MYA relates well to the mid-Miocene collision of the Sula-Spur with the Sundaland margin [1, 40, 43] (Fig 1E) and the resulting emergence of land in the paleo-Sulawesi archipelago. Ophiolite emplacements are indicative for areas of emergent land forming southeast Sulawesi from early Miocene onwards [40, 43], thus offering a conceivable point of arrival for the progenitor of Sulawesi tarsiers. However, paleo-geographic maps also indicate the existence of a small area of subaerial land in the region of today´s southwest peninsula of Sulawesi since the early Miocene [1, 38, 40] allowing for colonizing the island from this direction.

Our broad (although not all-encompassing) sampling of extant taxa suggests that, assuming no other yet undetected lineage split off before (see next section), diversification in crown Sulawesi tarsiers had begun at earliest 13 Ma after initial colonization of Sulawesi, most likely reflecting the emergence of scattered land masses in the region. At the Plio-Pleistocene border crown Sulawesi tarsiers split up into two lineages (Fig 1E). It is remarkable that this divergence event roughly corresponds to a glacial maximum at 2.5 MYA [19]. Assuming that tarsiers first populated Sulawesi´s south (either south-east or south-west), episodic exposure of shelves during the ice-age at 2.5 MYA may have enabled tarsiers to cross the Gulf of Bone [1, 13, 38, 44]. This in turn could have induced the formation of the two lineages, one expanding its range from southeast to north Sulawesi (lineage 1), and the other dispersing in a southwest—northeast direction (lineage 2, Fig 4).

The relatively deep divergence between southeastern and northern populations of lineage 1 might be the result of strike-slip faulting and the related formation of the Malili-Lake-System in southeast Sulawesi beginning in the Pliocene [45]. Assessing the phylogeographic structure in northern Sulawesi, our data did not clearly resolve evolutionary relationships between populations ranging from Ogatemuku (OGA) to the most northeastern tip of the island. Interglacial oceanic inundations, especially during the Pleistocene, are thought to have provoked allopatric speciation in several taxa inhabiting the northern peninsula [2–4]. However, sea-level induced range fragmentations may have been insufficently long-lasting for speciation to be completed in northern tarsier populations. Alternatively, reduced distribution ranges, either naturally or anthropogenically effected, or decreased population densities can promote interspecific hybridization [46, 47]. Relaxed assortative mating in order to avoid inbreeding among recently evolved tarsier populations inhabiting the narrow northern peninsula could therefore have led to the collapse of newly acquired reproductive barriers. A third option would be that extant populations of LAB and DUA are descendants from OGA and only recently colonized the central and most northeastern part of the northern peninsula.

Based on nuclear sequence data lineage 2-populations fall into two distinct clades, with *Tarsius lariang* being sister to *T*. *fuscus*, *T*. *dentatus* and the most eastern populations of KOR and LUW (Fig 1E). Based on our divergence time estimates we hypothesize two dispersal waves out of southwest Sulawesi, which was likely a separate island until mid-Pleistocene [48]. In this period (Proto-) *Tarsius lariang* could have reached west Sulawesi in a first wave (Fig 4). In a second wave, individuals may have colonized central parts of the island following an ecological gradient from dry to moist climatic conditions [49], which might have facilitated differentiation between *T*. *fuscus* and *T*. *dentatus*. Considering present cross-taxon congruence in endemism around Lake Tempe ([4], this study), recurrent flooding of the Tempe Depression during the Pleistocene [2] likely played a central role in driving and maintaining species divergence. In the course of fusing land masses, *T*. *dentatus* crossed the Palu-Koro fault [7] and superseded lineage 1-species from lowland rainforests in central and east Sulawesi (Figs 1C–1D and 4). Molecular evidence presented here confirms previous acoustic studies assigning tarsiers of the eastern peninsula to *T*. *dentatus* [9, 17]. In case of the still hybridizing tarsier species in central Sulawesi, the paleo-environmental setting around the Palu Valley seems to have maintained rather than initiated reproductive isolation [7] between the two recently diverged species *T*. *dentatus* and *T*. *lariang*.

Our reconstruction of a crown tarsiid phylogeny again strengthens a sister group relationship between Western and Philippine tarsiers suggesting their divergence in late Miocene [9, 50]. The median node age at 9.8 MYA perfectly matches the period of lowest tertiary sea level [1, 19, 51] possibly facilitating tarsier dispersal from Sundaland to Mindanao (Philippines) [52].

Aware that the use of secondary calibration points bears the risk of erroneous divergence time estimates [53], we are confident that relatively narrow confidence intervals, the obvious correspondence between tarsier divergence times and major geological events and climate fluctuations, as well as good node support for several sub-clades build a reliable framework for our inferences about tarsier diversification. Moreover, median node ages of crown tarsiers, the Western-/Philippine tarsier split and the initial speciation event in Sulawesi tarsiers are consistent with estimates by [9, 50] further strengthening the reliability of internal split times obtained in this study.

### Pathways to Sulawesi and Radiation to Mountain and Offshore Regions

Tarsiers likely crossed Wallace´s Line (see Fig 1A) taking advantage of episodically exposed dispersal corridors connecting the Sundaic region and the paleo-Sulawesi archipelago. Passages across the Makassar Strait and along the Sunda Arc cover the most conceivable routes (see Fig 1A). The Strait of Makassar forms a deep sea barrier between Borneo and Sulawesi since the Eocene. Intermittent land connections especially in its central regions are considered unlikely [41]. Further south temporarily emergent volcanic chains and carbonate platforms extending from Java over the East Java Sea to South Sulawesi could have served as stepping stones during Paleo-Neogene periods [1, 48, 54]. But, even though offering dispersal opportunities over geological times, the tectonic and paleoenvironmental instability of this region seems to have significantly decreased dispersal success of tarsiers. Our data suggest a single entry of a founder population into Sulawesi, likely going along with the separation of crown Sulawesi tarsiers from other crown tarsiers during the Miocene (see previous section). The long time gap between island colonization and diversification of Sulawesi tarsiers, however, allows alternative scenarios. In the first scenario, crown Sulawesi tarsiers made several attempts to colonize Sulawesi from the Proto-Java islands. One single lineage succeeded at the Plio-Pleistocene border and subsequently underwent further diversification while other lineages became extinct. In the second scenario, Tarsiers´ arrival on Sulawesi indeed falls into the Miocene epoch, but initial cladogenesis within Sulawesi tarsiers predates the corresponding bifurcation in our inferred species tree as a result of incomplete taxon sampling [55]. Although our sampling comprises a wide geographic coverage of Sulawesi lowland tarsiers, it lacks data on the montane pygmy tarsier [56] and on lowland species inhabiting some offshore islands near Sulawesi [16, 56–59] (Fig 4). We therefore cannot exclude insufficient taxon coverage as a possible source for the time gap between tarsier arrival and initial (observed) speciation on Sulawesi. However, it remains unclear at this point whether offshore and montane species are phylogenetically embedded within or evolved independently from the two major Sulawesi tarsier lineages described here.

### Units, Shifts, and Conservation

The Wallacea region is a hotspot of biodiversity [60]. However, as complex as its geological history is the spatial distribution of species on Sulawesi, the largest Wallacean island. Regional range overlaps of independent evolutionary lineages point to a shared biogeographic history, while at the same time highlighting discrepancies to other local species assemblages [8, 12, 61–64]. Nevertheless, there is a wide consensus for dispersal as the prevailing mode of island colonization—contrary to the hypothesis of ancient vicariance via micro-continental drift—and Plio-/Pleistocene diversification of most of Sulawesi´s extant terrestrial and limnic fauna [4, 7, 13, 14, 43, 63, 65] (see Results of this study). Cross-taxon congruence of distantly related terrestrial species like Sulawesi toads, macaques, and tarsiers (Fig 1C) [4] is strong evidence that at least parts of the island temporarily constituted isolated biotic entities in the more recent geological past. Current geographical and genetic structuring of Sulawesi tarsiers suggests that post-speciation range shifts may have moved species boundaries thus blurring positional signals of past reproductive isolation. Therefore, ancient (physical) barriers to gene flow do not necessarily qualify for defining general areas of conservation, although they provide useful guidance in identifying regions of genetic endemism [4]. Finally, further phylogeographic studies are needed to fully understand the history and progress of land formation on Sulawesi and its consequences for species diversity. Future work should in particular take advantage of powerful molecular tools rather than analyzing single (usually mitochondrial) genetic loci with higher susceptibility to incomplete lineage sorting, gender bias, and accelerated evolutionary rates that can lead to overestimates of divergence times [66–71] (S2 Text and S1 Fig).

## Supporting Information

S1 Fig Cytochrome b phylogenetic tree. Maximum likelihood phylogenetic inference based on mitochondrial cytochrome b haplotypes of Sulawesi tarsiers and evaluated by 1000 bootstrap replicates. Only nodes supported with bootstrap values above 700 are shown. Thick black branches mark cytochrome b haplotypes carried by individuals of the pruned sample set used for nuclear sequence-based species tree inference.
(TIF)

S2 Fig ABCA1 statistical parsimony network. Gap masking led to the collapsing of two distinct alleles (1: TLA; 2: shared by TDE and TFU) into one node (**). The black circle indicates an inferred missing haplotype. Mutation steps are shown as hatch marks.
(TIF)

S3 Fig ADORA3 statistical parsimony network. The black circles indicate inferred missing haplotypes. Mutation steps are shown as hatch marks.
(TIF)

S4 Fig AXIN1 statistical parsimony network. Mutation steps are shown as hatch marks.
(TIF)

S5 Fig RAG1 statistical parsimony network. Mutation steps are shown as hatch marks.
(TIF)

S6 Fig TTR statistical parsimony network. Gap masking led to the collapsing of two (**: 1 = KEN; 2 = KEN) respectively three distinct alleles (***: 1 = OGA; 2 = OGA, TWA, LAB, DUA; 3 = TLA) into one node.
(TIF)

S1 Table Primer and PCR information of phylogenetic loci. (DOCX)

S2 Table Models of nucleotide evolution. (DOCX)

S3 Table Sources of anthropoid and strepsirhine primate sequence data. (DOCX)

S4 Table Primate divergence times and node support. (DOCX)

S1 Text Laboratory procedures. (DOCX)

S2 Text Mitochondrial cytochrome b gene data base and analysis. (DOCX)
