# Supplementary figures and images for: Stop and Go – Waves of Tarsier Dispersal Mirror the Genesis of Sulawesi Island
Source: PLoS One. 2015 Nov 11;10(11):e0141212. doi: 10.1371/journal.pone.0141212 (PMC4641617; doi:10.1371/journal.pone.0141212)

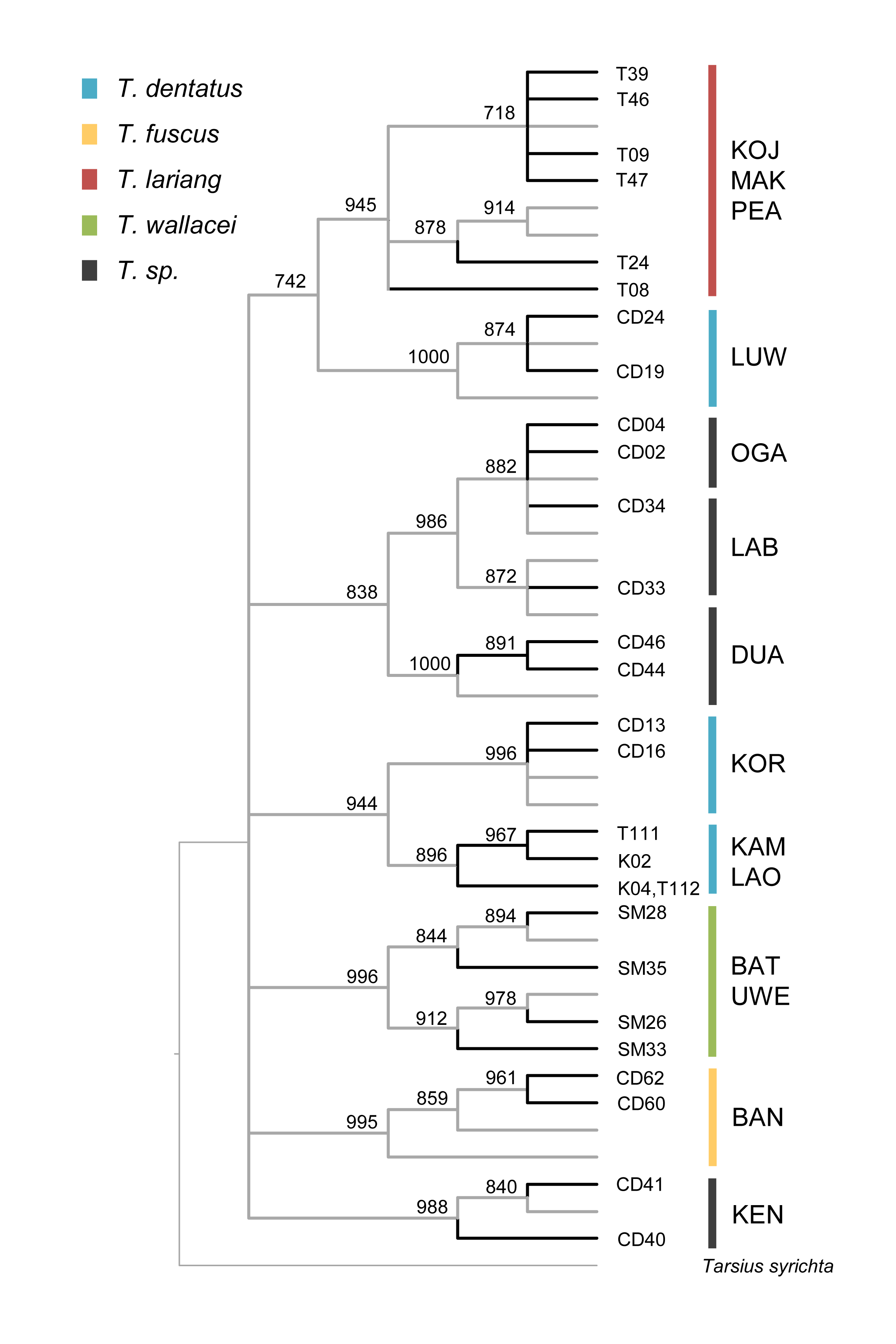

Supplement: S1 Fig — Maximum likelihood phylogenetic inference based on mitochondrial cytochrome b haplotypes of Sulawesi tarsiers and evaluated by 1000 bootstrap replicates. Only nodes supported with bootstrap values above 700 are shown. Thick black branches mark cytochrome b haplotypes carried by individuals of the pruned sample set used for nuclear sequence-based species tree inference. (TIF) [file pone.0141212.s001.tif]

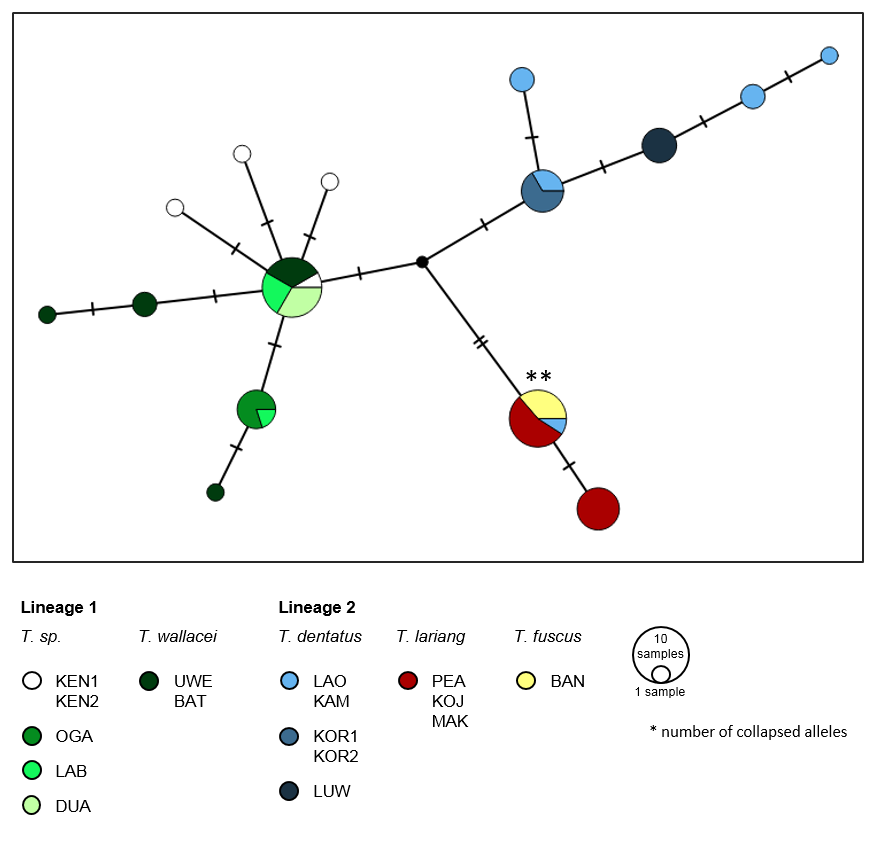

Supplement: S2 Fig — Gap masking led to the collapsing of two distinct alleles (1: TLA; 2: shared by TDE and TFU) into one node (**). The black circle indicates an inferred missing haplotype. Mutation steps are shown as hatch marks. (TIF) [file pone.0141212.s002.tif]

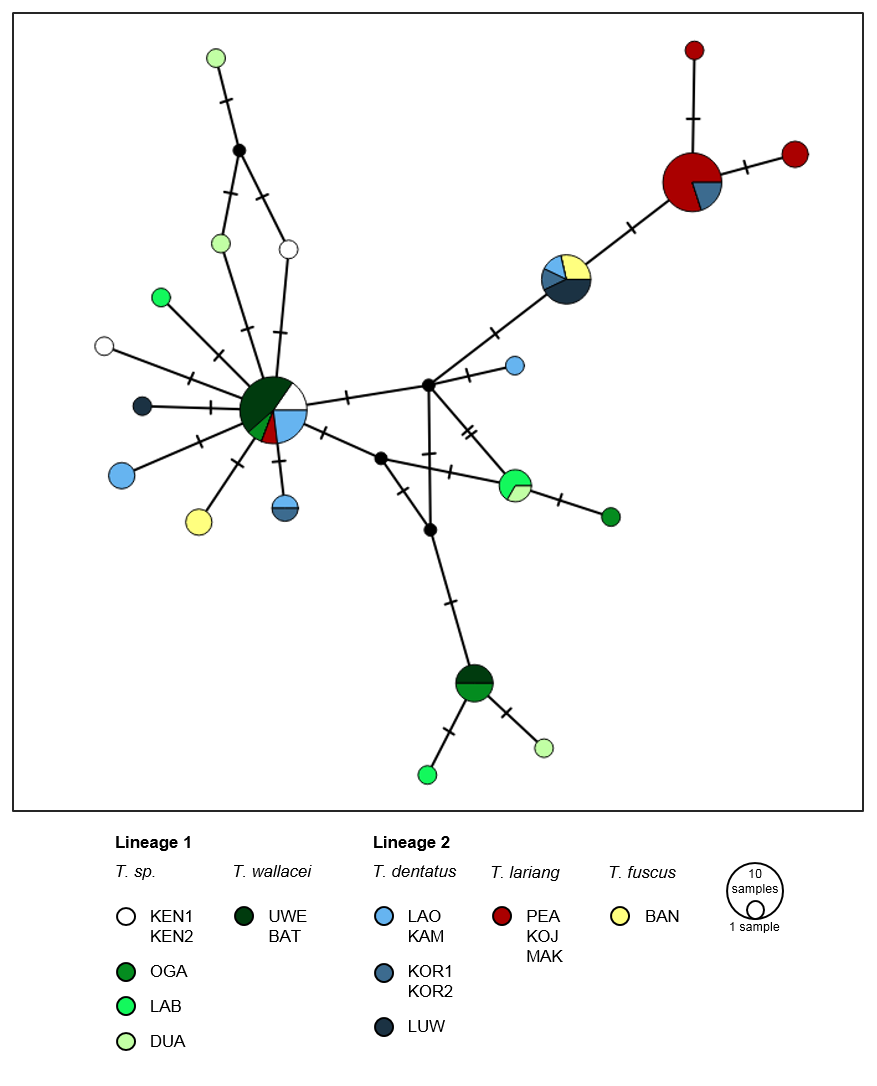

Supplement: S3 Fig — The black circles indicate inferred missing haplotypes. Mutation steps are shown as hatch marks. (TIF) [file pone.0141212.s003.tif]

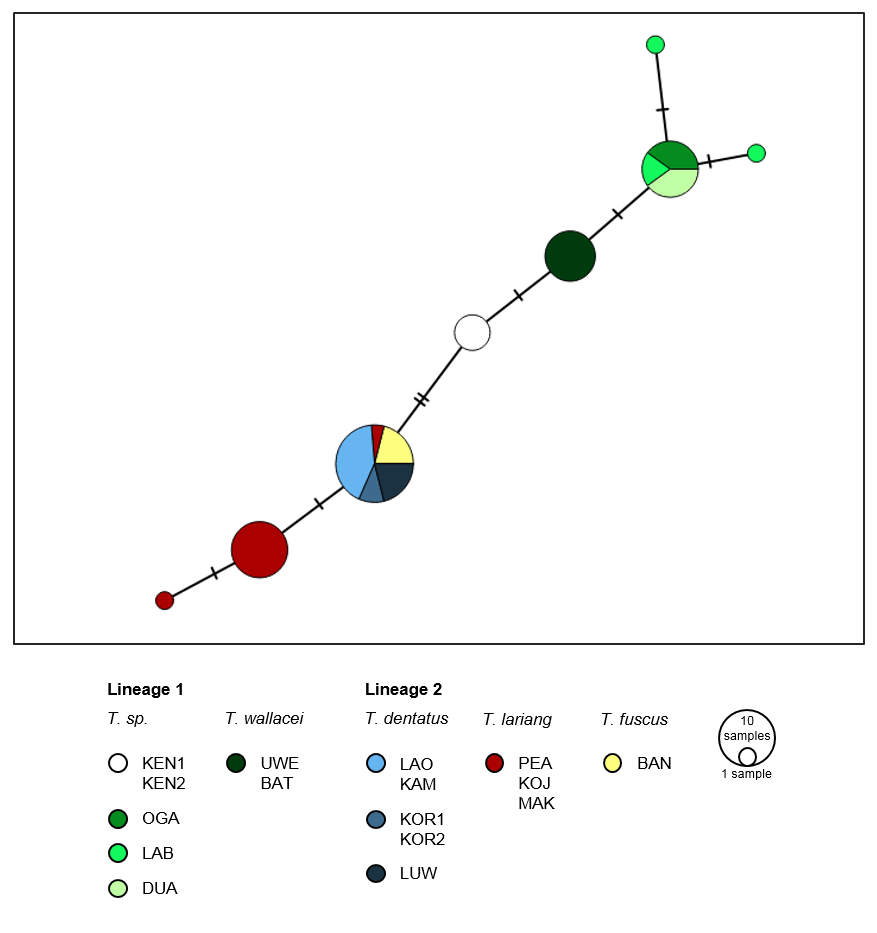

Supplement: S4 Fig — Mutation steps are shown as hatch marks. (TIF) [file pone.0141212.s004.tif]

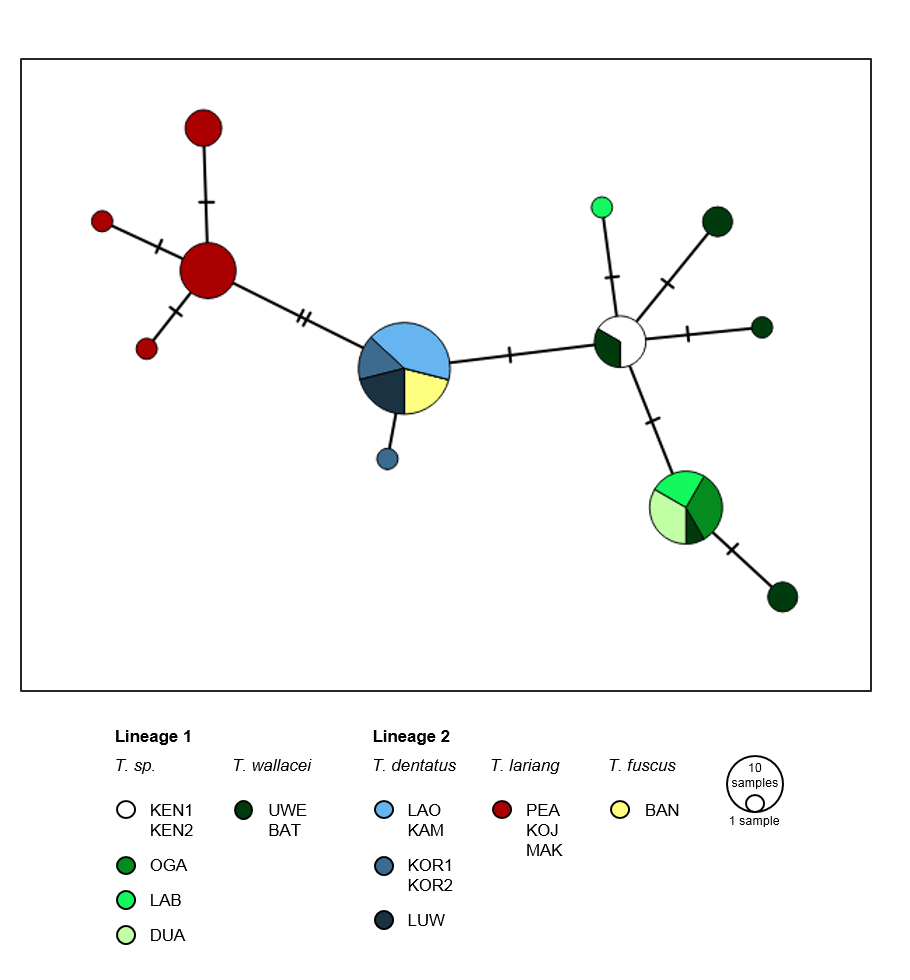

Supplement: S5 Fig — Mutation steps are shown as hatch marks. (TIF) [file pone.0141212.s005.tif]

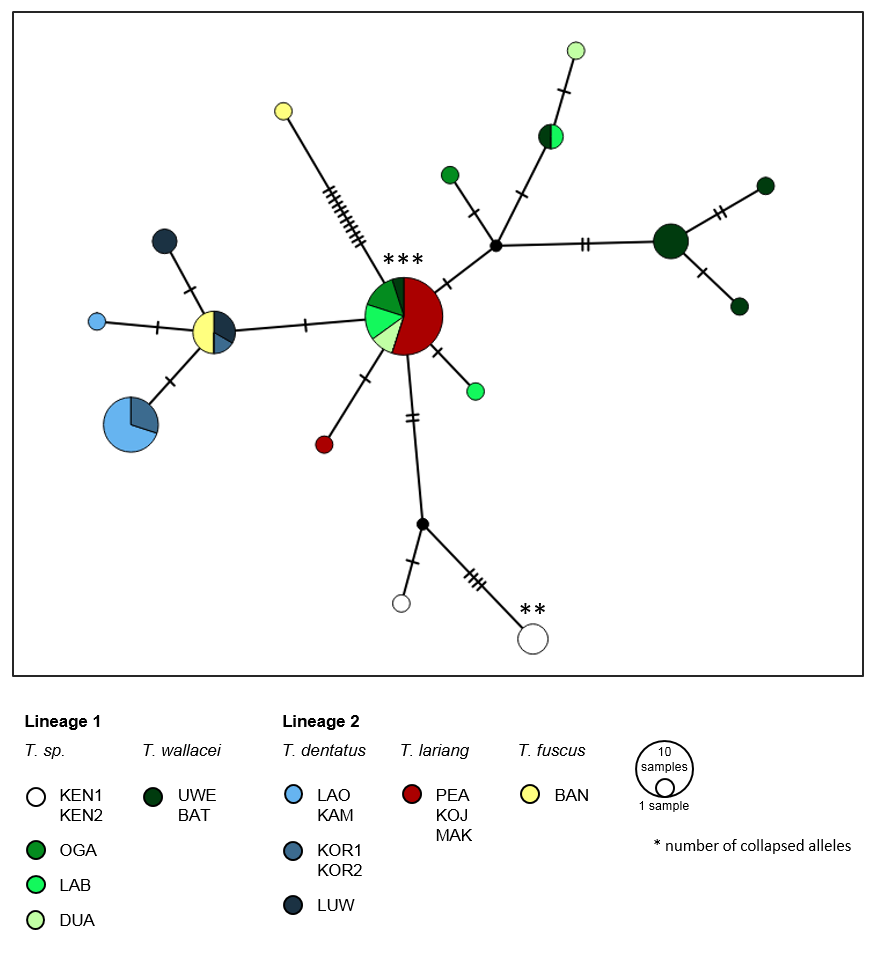

Supplement: S6 Fig — Gap masking led to the collapsing of two (**: 1 = KEN; 2 = KEN) respectively three distinct alleles (***: 1 = OGA; 2 = OGA, TWA, LAB, DUA; 3 = TLA) into one node. (TIF) [file pone.0141212.s006.tif]
