# Supplementary material for: Stop and Go – Waves of Tarsier Dispersal Mirror the Genesis of Sulawesi Island
Source: PLoS One. 2015 Nov 11;10(11):e0141212. doi: 10.1371/journal.pone.0141212 (PMC4641617; doi:10.1371/journal.pone.0141212)
Supplement: S1 Table — (DOCX) [file pone.0141212.s007.docx]

# S1 Table. ****Primer and PCR information of phylogenetic loci.****

| **Locus** | **Gene** | **Target** | **Forward primer sequence 5'-3' /** | **Amplicon** | **Target** | **T_A_^b^** | **Ext^c^** | **Primer Reference** |
| --- | --- | --- | --- | --- | --- | --- | --- | --- |
|  |  | **Position^a^** | **Reverse primer sequence 5'-3'** | **Size (bp)** | **Size (bp)** | **(°C)** | **(mm:ss)** |  |
| ABCA1 | ATP-binding cassette sub-family A member 1 | intronic | CCTCCATCTTTTCAGCTCTACCTAC / | 645-651 | 535-536 | 59 | 01:00 | Horvath et al. 2008^[[1]](#footnote-1)^ |
|  |  |  | ACAAGAGCCTGGAGATTGGATAAC |  |  |  |  |  |
| ADORA3 | Adenosine receptor A3 | exonic | ACCCCCATGTTTGGCTGGAA / | 411 | 370 | 58 | 00:45 | Murphy et al. 2001^[[2]](#footnote-2)^ |
|  |  |  | GATAGGGTTCATCATGGAGTT |  |  |  |  |  |
| AXIN1 | Axin 1 isoform a | exonic | CTCTGCCTTCGCTGTACCGTCTAC / | 995 | 809 | 58 | 01:00 | Horvath et al. 20081 |
|  |  |  | CCCACCTTTCCTAATCCTTGTCCTC^d^ |  |  |  |  |  |
| RAG1 | Recombination activating gene 1 | exonic | AAGACATCCTGGAAGGCATGA^e^ / | 845 | 745 | 58 | 01:00 | Murphy et al. 2001^2^ |
|  |  |  | AAAGTTGCCGTTCATCCTCA^e^ |  |  |  |  |  |
| TTR | Thyroxine-binding prealbumin | intronic | TGCCTTGCTGGACTGGTATT^e^ / | 1005-1025 | 891-911 | 58 | 01:00 | Flynn and Nedbal 1998^[[3]](#footnote-3)^ |
|  |  |  | GACGGCATCTAGTACTTTGACCAT^f^ |  |  |  |  |  |

^a^ Based on the human genome

^b^ Annealing temperature

^c^ Extension time

Primer modifications (based on *T. syrichta* sequences of the Ensembl Genome database):

^d^ Primer sequence modified from Horvath et al. 2008

^e^ 1 mismatch to the published primer sequence

^f^ 2 mistmatches to the published primer sequence

1. Horvath JE, Weisrock DW, Embry SL, Fiorentino I, Balhoff JP, Kappeler P, et al. Development and application of a phylogenomic toolkit: Resolving the evolutionary history of Madagascar´s lemurs. Genome Res. 2008; 18: 489-499. [↑](#footnote-ref-1)
2. Murphy WJ, Eizirik E, Johnson WE, Zhang YP, Ryder O., O´Brien SJ. Molecular phylogenetics and the origins of placental mammals. Nature. 2001; 409: 614-618. [↑](#footnote-ref-2)
3. Flynn JJ, Nedbal MA. Phylogeny of the Carnivora (Mammalia): Congruence vs incompatibility among multiple data sets. Mol Phylogenet Evol. 1998; 9: 414-426. [↑](#footnote-ref-3)
