# Supplementary material for: Stop and Go – Waves of Tarsier Dispersal Mirror the Genesis of Sulawesi Island
Source: PLoS One. 2015 Nov 11;10(11):e0141212. doi: 10.1371/journal.pone.0141212 (PMC4641617; doi:10.1371/journal.pone.0141212)
Supplement: S2 Table — (DOCX) [file pone.0141212.s008.docx]

# ****S2 Table. Models of nucleotide evolution.****

| **Locus** | **Gene tree^a^** | **Species tree^a^** | **Calibrated species tree^b^** |
| --- | --- | --- | --- |
| ABCA1 |  | HKY | TVM |
| ADORA3 |  | HKY+I | J1+G (4 cat) |
| AXIN1 |  | HKY | HKY+G (4 cat) |
| RAG1 |  | TN+I | J1+G (4cat) |
| TTR |  | HKY+G (4 cat) | TVM+G (4cat) |
| SRY | TN |  |  |

Species and gene trees inferred from sequence data including tarsiers (^a^) or representatives of haplorhine and strepsirhine primates (^b^, Table S4). Proposed best-fit substitution models based on Akaike´s information criterion corrected for small sample sizes (AICc) using Treefinder v. March 2011 ^[[1]](#footnote-1)^. HKY: Hasegawa, Kishino and Yano model ^[[2]](#footnote-2)^; TN: Tamura-Nei two parameter model ^[[3]](#footnote-3)^; TVM: Transversion model ^[[4]](#footnote-4)^; J1: Transition model ^[[5]](#footnote-5)^; I: Invariable sites model; G: Discrete Gamma model; cat: rate categories.

1. Jobb G, von Haeseler A, Strimmer K. TREEFINDER: a powerful graphical analysis environment for molecular phylogenetics. BMC Evol Biol. 2004; 4: 18. [↑](#footnote-ref-1)
2. Hasegawa M, Kishino H, Yano T. Dating of the human-ape splitting by a molecular clock of mitochondrial DNA. J. Mol Evol. 1985; 22: 160-174. [↑](#footnote-ref-2)
3. Tamura K, Nei M. Estimation of the number of nucleotide substitutions in the control region of mitochondrial DNA in humans and chimpanzees. Mol Biol Evol. 1993; 10: 512-526. [↑](#footnote-ref-3)
4. Rodriguez F, Oliver JL, Marin A, Medina JR. The general stochastic model of nucleotide substitution. J Theor Biol. 1990; 142: 485-501. [↑](#footnote-ref-4)
5. Posada D. jModelTest: Phylogenetic model averaging. Mol Biol Evol. 2008; 25: 1253-1256. [↑](#footnote-ref-5)
