# Supplementary material for: Stop and Go – Waves of Tarsier Dispersal Mirror the Genesis of Sulawesi Island
Source: PLoS One. 2015 Nov 11;10(11):e0141212. doi: 10.1371/journal.pone.0141212 (PMC4641617; doi:10.1371/journal.pone.0141212)
Supplement: S3 Table — (DOCX) [file pone.0141212.s009.docx]

# ****S3 Table. Sources of anthropoid and strepsirhine primate sequence data.****

| **Taxon** | **Gene** | **Sequence** | **Source** |
| --- | --- | --- | --- |
| *Callithrix* | ABCA1 | HM765296 | GenBank |
|  |  | chromosome:C_jacchus3.2.1:1:148170393:148191518:1 | Ensembl |
|  | ADORA3 | HM765164 | GenBank |
|  |  | chromosome:C_jacchus3.2.1:7:147463921:147464931:-1 | Ensembl |
|  | AXIN1 | HM765306 | GenBank |
|  |  | chromosome:C_jacchus3.2.1:12:351493:353038:-1 | Ensembl |
|  | RAG1 | HM759090 | GenBank |
|  |  | chromosome:C_jacchus3.2.1:11:99861889:99868965:1 | Ensembl |
|  | TTR | HM757710 | GenBank |
|  |  | AY434071^a^ | GenBank |
| *Cheirogaleus* | ABCA1 | EU057428 | GenBank |
|  | ADORA3 | EU342218 | GenBank |
|  | AXIN1 | HM764359 | GenBank |
|  | RAG1 | HM759144 | GenBank |
|  | TTR | AY434064^a^ | GenBank |
| *Daubentonia* | ABCA1 | EU057429 | GenBank |
|  | ADORA3 | EU342219 | GenBank |
|  | AXIN1 | EU057284 | GenBank |
|  | RAG1 | EU342306 | GenBank |
|  | TTR | EU342331 | GenBank |
| *Homo* | ABCA1 | HM765327 | GenBank |
|  |  | NG_007981 | GenBank |
|  | ADORA3 | HM765141 | GenBank |
|  |  | NG_032119 | GenBank |
|  | AXIN1 | HM764284 | GenBank |
|  |  | NG_012267 | GenBank |
|  | RAG1 | HM759069 | GenBank |
|  |  | NG_007528 | GenBank |
|  | TTR | HM757691 | GenBank |
|  |  | NG_009490 | GenBank |
| *Hylobates* | ABCA1 | HM765324 | GenBank |
|  | ADORA3 | HM765143 | GenBank |
|  | AXIN1 | HM764286 | GenBank |
|  | RAG1 | HM759071 | GenBank |
|  | TTR | HM757693 | GenBank |
| *Macaca* | ABCA1 | HM757659 | GenBank |
|  |  | chromosome:MMUL_1:15:31274263:31325078:-1 | Ensembl |
|  | ADORA3 | HM757659 | GenBank |
|  |  | chromosome:MMUL_1:1:114504821:114508531:-1 | Ensembl |
|  | AXIN1 | HM757659 | GenBank |
|  |  | chromosome:MMUL_1:20:334373:345620:1 | Ensembl |
|  | RAG1 | HM757659 | GenBank |
|  |  | AY011900^b^ | GenBank |
|  | TTR | HM757659 | GenBank |
|  |  | FJ846620^c^ | GenBank |
| *Otolemur* | ABCA1 | EU057451 | GenBank |
|  | ADORA3 | EU342237 | GenBank |
|  | AXIN1 | HM764378 | GenBank |
|  | RAG1 | HM759164 | GenBank |
|  | TTR | scaffold:OtoGar3:GL873531.1:19802889:19810218:-1 | Ensembl |
| *Pan* | ABCA1 | HM765384 | GenBank |
|  |  | HM765385 | GenBank |
|  | ADORA3 | HM765152 | GenBank |
|  |  | HM765153 | GenBank |
|  | AXIN1 | HM764294 | GenBank |
|  |  | HM764295 | GenBank |
|  | RAG1 | HM759079 | GenBank |
|  |  | chromosome:CHIMP2.1.4:11:36563611:36570686:1 | Ensembl |
|  | TTR | HM757700 | GenBank |
|  |  | HM757701 | GenBank |
| *Pongo* | ABCA1 | HM765381 | GenBank |
|  |  | HM765382 | GenBank |
|  | ADORA3 | HM765154 | GenBank |
|  |  | HM765155 | GenBank |
|  | AXIN1 | HM764296 | GenBank |
|  |  | HM764297 | GenBank |
|  | RAG1 | HM759081 | GenBank |
|  |  | HM759082 | GenBank |
|  | TTR | HM757702 | GenBank |
|  |  | HM757703 | GenBank |

Blue: Perelman et al. (2001)^[[1]](#footnote-1)^; red: Horvath et al. (2008)^[[2]](#footnote-2)^; green: NCBI RefSeqGene;

a: Yoder and Yang (2004)^[[3]](#footnote-3)^; b:Murphy et al. (2001); c: Stevison and Kohn (2009)^[[4]](#footnote-4)^.

1. Perelman P, Johnson WE, Roos C, Seuánez HN, Horvath JE, Moreira MAM, et al. A Molecular Phylogeny of Living Primates. PLoS Genet. 2011; 7: e1001342. [↑](#footnote-ref-1)
2. Horvath JE, Weisrock DW, Embry SL, Fiorentino I, Balhoff JP, Kappeler P, et al. Development and application of a phylogenomic toolkit: Resolving the evolutionary history of Madagascar´s lemurs. Genome Res. 2008; 18: 489-499. [↑](#footnote-ref-2)
3. Yoder AD, Yang Z. Divergence dates for Malagasy lemurs estimated from multiple gene loci: geological and evolutionary context. Mol Ecol. 2004; 13: 757-773. [↑](#footnote-ref-3)
4. Stevison LS, Kohn MH. Divergence population genetic analysis of hybridization between rhesus and cynomolgus macaques. Mol Ecol 2009; 18: 2457-2475. [↑](#footnote-ref-4)
