# Supplementary material for: Stop and Go – Waves of Tarsier Dispersal Mirror the Genesis of Sulawesi Island
Source: PLoS One. 2015 Nov 11;10(11):e0141212. doi: 10.1371/journal.pone.0141212 (PMC4641617; doi:10.1371/journal.pone.0141212)
Supplement: S4 Table — (DOCX) [file pone.0141212.s010.docx]

# ****S4 Table. Primate divergence times and node support.****

| **Node** | **median** | **95 % HPD^a^** |  | **pp^b^** |
| --- | --- | --- | --- | --- |
|  | **node age (MYA)** | **lower** | **upper** |  |
| **Homininae** | 4.12 | 1.07 | 6.58 | 1.00 |
| **Hominidae** | 16.68 | 14.03 | 18.76 | 0.93 |
| Hominoidea | 18.68 | 15.88 | 21.68 | 1.00 |
| **Catharrini** | 29.32 | 25.10 | 33.36 | 1.00 |
| **Anthropoidea** | 42.11 | 37.48 | 46.66 | 1.00 |
| **Haplorhini** | 84.34 | 76.10 | 93.02 | 0.97 |
| **Strepsirhini** | 66.36 | 58.60 | 73.90 | 1.00 |
| Lemuriformes | 54.81 | 45.92 | 64.03 | 1.00 |
| **Primates** | 90.68 | 83.34 | 98.17 | 1.00 |

Calibrated nodes are written in bold. ^a^ Lower and upper bound of the highest posterior density;

^b^posterior probability.
