# Supplementary material for: Stop and Go – Waves of Tarsier Dispersal Mirror the Genesis of Sulawesi Island
Source: PLoS One. 2015 Nov 11;10(11):e0141212. doi: 10.1371/journal.pone.0141212 (PMC4641617; doi:10.1371/journal.pone.0141212)
Supplement: S1 Text — (DOCX) [file pone.0141212.s011.docx]

# S1 Text. Laboratory procedures.

Wax-mediated hot-start PCR amplifications of mitochondrial and nuclear gene markers were carried out in 30 µl reaction volumes composed of 10x PCR buffer (containing 15 mM MgCl2), 200 μM of each dNTP, and 0.75 U of Taq DNA polymerase from the Qiagen Taq PCR Core Kit, 0.33 μM of each primer, and 20-40 ng DNA template. PCRs were conducted under the following thermal cycling conditions: Initial denaturation at 94°C for 3 min followed by 35 cycles of denaturation (94 °C for 40 sec), annealing (1 min, see Table S2 for primer-specific annealing temperatures), and extension (see Table S2 for specific extension times). PCR products were cleaned up by Exonuclease I and Shrimp Alkaline Phosphatase (Fermentas) treatment and sequenced in 10 µl reaction volumes using the Big Dye® Terminator v. 3.1 Cycle Sequencing Kit. Cycle sequencing reactions contained 1 μl Big Dye premix, 2 μl 5x Sequencing buffer, and 1 μl Primer (10 pmol/μl). The cycle sequencing conditions were as follows: 5 min denaturation step at 96 °C, followed by 30 cycles of 96 °C for 10 sec and 4 min at primer specific temperatures. Unincorporated dye terminators were scavenged by SDS/heat treatment.

PCR products from individuals which were heterozygous for a biparental inherited gene were purified by ethanol precipitation and cloned using the pGEM®-T vector system I (Promega). After ligation products were extracted with phenol/chloroform purification and ethanol precipitation. Resolved plasmid DNA was electroporated into competent One Shot® TOP10 E. coli cells (Invitrogen). Transformed cells were grown at 37 °C for 1 hour on a shaker, plated onto ampicillin-selective LB agar plates, and incubated at 37 °C overnight. At least eight colonies were selected after blue/white screening and amplified in 20 µl reaction volumes using a standard PCR protocol and the Qiagen Taq PCR Core Kit (concentrations of reaction components and thermal cycling profiles correspond with those of the hot start PCR).
