# Supplementary material for: Stop and Go – Waves of Tarsier Dispersal Mirror the Genesis of Sulawesi Island
Source: PLoS One. 2015 Nov 11;10(11):e0141212. doi: 10.1371/journal.pone.0141212 (PMC4641617; doi:10.1371/journal.pone.0141212)
Supplement: S2 Text — (DOCX) [file pone.0141212.s012.docx]

**S2 Text. Mitochondrial cytochrome b gene data base and analysis.**

We PCR-amplified and sequenced the cytochrome b gene of 65 tarsier individuals sampled in the framework of this study (Genebank accession numbers KR337026-KR337090) adopting PCR primers and conditions from Merker et al. (2009)^[[1]](#footnote-1)^. Data sets of previous studies^1^^[[2]](#footnote-2)^^[[3]](#footnote-3)^ comprising 86 individuals of Eastern tarsiers were also included in the tree reconstruction (Genbank accession numbers FJ214312-FJ214337, FJ614263-FJ614285, FJ614291-FJ614297, FJ614300-FJ614306, FJ614364-FJ614371, HM115970-HM115984). *Tarsius syrichta* served as outgroup (Genbank accession number AB371090).

We estimated a maximum likelihood phylogeny of 44 cytochrome b haplotypes (S1 Fig.) obtained from 151 individuals using PhyML-aBayes^[[4]](#footnote-4)^^[[5]](#footnote-5)^ with the substitution model J3 and gamma distribution with five categories selected as described in the main text. Bootstrap support was assessed by 1000 replicates.

We preferred nuclear compared to matrilinear inherited DNA data for phylogenetic analyses to obtain reliable inferences about phylogenetic relationships and populations divergence times -mtDNA evolves more rapidly than nuclear DNA^[[6]](#footnote-6)^- and to minimize gender-biased admixture^[[7]](#footnote-7)^, as we found strong indication for female philopatry and male dispersal (single-male multi-female groups at most study sites; nearly all cytochrome b haplotypes were unique to a single study locality).

1. Merker S, Driller C, Perwitasari-Farajallah D, Pamungkas J, Zischler H. Elucidating geological and biological processes underlying the diversification of Sulawesi tarsiers. Proc Natl Acad Sci USA. 2009; 106: 8459-8464. [↑](#footnote-ref-1)
2. Driller C, Perwitasari-Farajallah D, Zischler H, Merker S. The social system of Lariang tarsiers (*Tarsius lariang*) as revealed by genetic analyses. Int J Primatol. 2009; 30: 267–281. [↑](#footnote-ref-2)
3. Merker S, Driller C, Dahruddin H, Wirdateti, Sinaga W, Perwitasari-Farajallah D, et al. Tarsius wallacei: a new tarsier species from Central Sulawesi occupies a discontinuous range. Int J Primatol. 2010; 31: 1107-1122. [↑](#footnote-ref-3)
4. Guindon S, Gascuel O. A simple, fast, and accurate algorithm to estimate large phylogenies by maximum likelihood. Systematic Biology. 2003; 52:696-704. [↑](#footnote-ref-4)
5. Guindon S, Dufayard JF, Lefort V, Anisimova M, Hordijk W, Gascuel O. New algorithms and methods to estimate maximum-likelihood phylogenies: assessing the performance of PhyML 3.0. Systematic Biology. 2010; 59:307-321. [↑](#footnote-ref-5)
6. Vawter L, Brown WM. Nuclear and mitochondrial DNA comparisons reveal extreme rate variation in the molecular clock. Science. 1986; 234: 194-196. [↑](#footnote-ref-6)
7. Melnick DJ, Hoelzer GA. Differences in male and female macaque dispersal lead to contrasting distributions of nuclear and mitochondrial DNA variation. Int J Primatol. 1992; 13: 379-393. [↑](#footnote-ref-7)
